# Mitochondrial DNA Copy Number as a Biomarker for Guiding Adjuvant Chemotherapy in Stages II and III Colorectal Cancer Patients with Mismatch Repair Deficiency: Seeking Benefits and Avoiding Harms

**DOI:** 10.1245/s10434-024-15759-y

**Published:** 2024-07-10

**Authors:** Mian Chen, Shenghe Deng, Yinghao Cao, Jun Wang, Falong Zou, Junnang Gu, Fuwei Mao, Yifan Xue, Zhenxing Jiang, Denglong Cheng, Ning Huang, Liang Huang, Kailin Cai

**Affiliations:** 1 Department of Gastrointestinal Surgery, Union Hospital, Tongji Medical College, Huazhong University of Science and Technology, Wuhan, Hubei, China; 2 Department of General Surgery (Colorectal Surgery), The Sixth Affiliated Hospital, Sun Yat-sen University, Guangdong, Guangzhou, China; 3 Cancer Center, Union Hospital, Tongji Medical College, Huazhong University of Science and Technology, Wuhan, Hubei, China; 4 Hubei Key Laboratory of Biological Targeted Therapy, Union Hospital, Tongji Medical College, Huazhong University of Science and Technology, Wuhan, Hubei, China

**Keywords:** Colorectal cancer, Mismatch repair deficient, Adjuvant chemotherapy, Mitochondrial DNA copy number

## Abstract

**Background:**

Colorectal cancer (CRC) patients with mismatch repair-deficient/microsatellite instability-high (dMMR/MSI-H) status are conventionally perceived as unresponsive to adjuvant chemotherapy (ACT). The mitochondrial transcription factor A (TFAM) is required for mitochondrial DNA copy number (mtDNA-CN) expression. In light of previous findings indicating that the frequent truncating-mutation of TFAM affects the chemotherapy resistance of MSI CRC cells, this study aimed to explore the potential of mtDNA-CN as a predictive biomarker for ACT efficacy in dMMR CRC patients.

**Methods:**

Levels of MtDNA-CN were assessed using quantitative real-time polymerase chain reaction (qRT-PCR) in a cohort of 308 CRC patients with dMMR comprising 180 stage II and 128 stage III patients. Clinicopathologic and therapeutic data were collected. The study examined the association between mtDNA-CN levels and prognosis, as well as the impact of ACT benefit on dMMR CRC patients. Subgroup analyses were performed based mainly on tumor stage and mtDNA-CN level. Kaplan-Meier and Cox regression models were used to evaluate the effect of mtDNA-CN on disease-free survival (DFS) and overall survival (OS).

**Results:**

A substantial reduction in mtDNA-CN expression was observed in tumor tissue, and higher mtDNA-CN levels were correlated with improved DFS (73.4% vs 85.7%; *P* = 0.0055) and OS (82.5% vs 90.3%; *P* = 0.0366) in dMMR CRC patients. Cox regression analysis identified high mtDNA-CN as an independent protective factor for DFS (hazard ratio [HR] 0.547; 95% confidence interval [CI] 0.321–0.934; *P* = 0.0270) and OS (HR 0.520; 95% CI 0.272–0.998; *P* = 0.0492). Notably, for dMMR CRC patients with elevated mtDNA-CN, ACT significantly improved DFS (74.6% vs 93.4%; *P* = 0.0015) and OS (81.0% vs 96.7%; *P* = 0.0017), including those with stage II or III disease.

**Conclusions:**

The mtDNA-CN levels exhibited a correlation with the prognosis of stage II or III CRC patients with dMMR. Elevated mtDNA-CN emerges as a robust prognostic factor, indicating improved ACT outcomes for stages II and III CRC patients with dMMR. These findings suggest the potential utility of mtDNA-CN as a biomarker for guiding personalized ACT treatment in this population.

**Supplementary Information:**

The online version contains supplementary material available at 10.1245/s10434-024-15759-y.

The incidence of colorectal cancer (CRC) has consistently risen, resulting in significant societal burdens.^1^ Primary treatment for stages II and III CRC after surgery involves 5-fluorouracil (5-FU)-based adjuvant chemotherapy (ACT).^2,3^ However, not all CRC patients derive benefits from ACT.^4,5^

Approximately 10 to 20% of CRC cases exhibit mismatch repair-deficient/microsatellite instability-high (dMMR/MSI-H) status,^6^ stemming from DNA MMR gene inactivation (MLH1, MSH2, MSH6, and PMS2).^7^ Numerous studies have reported diminished or absent responses to fluorouracil-based ACT in dMMR CRC patients versus CRC patients with mismatch repair-proficient/microsatellite-stable/(pMMR/MSS).^8,9^

Conversely, a survival advantage has been observed for stage III CRC patients with dMMR when treated with a combination of oxaliplatin and fluorouracil.^10,11^ Nevertheless, some studies argue against the definitive survival benefit of oxaliplatin for dMMR CRC patients.^12,13^ Despite extensive research, the molecular mechanisms underlying chemotherapy insensitivity in dMMR CRC patients remain unclear. In response to this, the National Comprehensive Cancer Network (NCCN) guidelines currently advocate for sparing ACT for stage II CRC patients with dMMR/MSI-H due to inefficacy, whereas all stage III CRC patients are to receive ACT regardless of MMR status.^14^ Given the substantial associated costs, toxicity, and inconvenience of inappropriate treatments, there is a pressing need to identify predictive biomarkers for ACT efficacy in CRC patients with dMMR.

Mitochondrial DNA copy number (mtDNA-CN), a marker of mitochondrial function,^15^ is linked to development of chemoresistance in various malignancies.^16,17^ Prior studies have indicated that decreased mtDNA-CN contributes to chemotherapy resistance in ovarian cancer and esophageal squamous cell carcinoma.^17,18^ Moreover, human MSI-H CRC frequently exhibits truncating mutations in mitochondrial transcription factor A (TFAM), impairing mitochondrial stability and augmenting cisplatin-dependent apoptotic resistance.^19^ As TFAM regulates mtDNA-CN expression, TFAM-truncating mutations result in decreased mtDNA-CN expression.^19,20^ Therefore, it is hypothesized that mtDNA-CN could function as a predictive biomarker for ACT efficacy in dMMR CRC patients.

Based on this, we conducted the current study to assess the impact of mtDNA-CN on prognosis and ACT efficacy in dMMR CRC patients. To our knowledge, this report marks the first instance of mtDNA-CN proposed as an efficacy-predictive biomarker for ACT treatment of dMMR CRC patients.

## Methods

### Patient Samples

This study collected or obtained 308 CRC specimens with dMMR tissue, including 180 stage II cases and 128 stage III cases, from the tumor tissue bank of the hospital. The selection criteria specified CRC patients who underwent radical resection and patients with pathologic staging of stage II or III disease identified as dMMR by immunohistochemistry (IHC). The exclusion criteria ruled out patients who had local excisions performed, patients who had a history of malignant tumors, and patients with multiple primary CRCs.

**Fig. 1:**
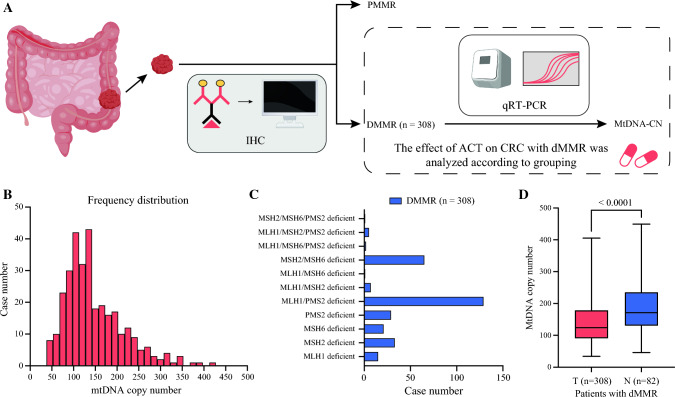
Research flow chart and mtDNA-CN characteristics of stages II and III CRC patients with dMMR. **A** Schematic diagram of research flow. **B** The distribution of mtDNA-CN in CRC cases. **C** The deficient mismatch repair proteins in 308 locally advanced colorectal cancer patients. **D** The mtDNA-CN ratio in tumor tissue versus non-tumor tissues cases. MtDNA-CN, mitochondrial DNA copy number; CRC, colorectal cancer; dMMR, deficient mismatch repair; IHC, immunohistochemistry; PMMR, mismatch repair-proficient; qPT-PCR, quantitative real-time polymerase chain reaction; ACT, adjuvant chemotherapy; T, tumor; N, non-cancerous

The clinicopathologic information and follow-up data of the patients came from the hospital database. The research contents and procedures were approved by the Institutional Review Committee of the Sixth Affiliated Hospital of Sun Yat-sen University (project no. E2021156). All patient information was treated anonymously and confidentially, so informed consent was waived. The analysis process is shown in Fig. 1A.

Long-term follow-up evaluation was performed every 6 months for all the patients by telephone or re-examination, and their relevant information was recorded. The main evaluation methods included clinical physical examination, imaging examination, and carcinoembryonic antigen (CEA) detection. In addition, it was recommended that the patients receive computed tomography and colonoscopy every year. Disease-free survival (DFS) was defined as the duration from radical resection to the last follow-up date or tumor recurrence, and overall survival (OS) was defined as the duration from radical resection to the last follow-up date or death from any cause. These follow-up evaluations continued until the death of the patient or the end of the study follow-up period (May 2023).

### DNA Isolation

The DNA isolation kit (Omega, Norcrosss, GA, USA) was used to extract DNA from tumor and normal tissues. The specific experimental steps were carried out according to the method provided in the kit protocol, and 30 mg of tissue was cut from each specimen for genomic DNA separation. Moreover, the purity of DNA samples was assessed by Nanodrop technology, and unqualified DNA samples were re-extracted. Finally, all qualified DNA samples were included for the next mtDNA-CN analysis.

### Determination of mtDNA-CN

For the determination of mtDNA-CN, quantitative real-time polymerase chain reaction (qPT-PCR) was performed using the Applied Biosystems 7500 Fast Real-Time PCR System (Thermo Fisher Scientific, Waltham, MA, USA). According to the qPT-PCR protocol of TB Green Premix Ex Taq II (TaKaRa, Shiga, Japan), the DNA input of each sample was 100 ng, and each sample was measured three times to ensure reliability of the results. The mean value was used as the mtDNA-CN score for each individual. The primer sequences used were hemoglobin (HGB)-F, 5′-GTGCACCTGACTCCTGAGGAGA-3′ and HGB-R, 5′-CCTTGATACCAACCTGCCCAG-3′ for hemoglobin (nuclear DNA control) and ND1-F, 5′-CCCTAAAACCCGCCACATCT-3′ and ND1-R, 5′-GAGCGATGGTGAGAGCTAAGGT-3′ for mitochondrial NADH dehydrogenase 1, respectively. The qRT-PCR reactions were performed at 95 °C for 30 s followed by 40 cycles of 95 °C for 5 s and 60 °C for 30 s. Based on the threshold cycle value (Ct values), the MtDNA-CN of each sample was calculated by the 2^-ΔCT^ method.

### Treatment

For the patients who received neoadjuvant chemoradiotherapy, from Monday to Friday, the tumor and entire pelvis were treated with radiotherapy (23–25 doses), for a total dose of 46.0 to 50.4 Gy. The patients received preoperative therapy with four to six cycles of fluorouracil (folinate 400 mg/m^2^ intravenous infusion, fluorouracil 400 mg/m^2^ intravenous infusion, fluorouracil 2.4 g/m^2^ intravenous continuous infusion for 48 h) and oxaliplatin 85 mg/m^2^ intravenously each chemotherapy treatment cycle, as well as radiotherapy during the two to four cycles of chemoradiotherapy. After surgery, six to eight cycles of mFOLFOX6 chemotherapy were administered to patients who required ACT, with additional radiotherapy at the physician's discretion.

### Pathologic Assessment

Two experienced pathologists were responsible for the relevant evaluation of surgical pathologic specimens. Immunohistochemistry (IHC) was used to evaluate the MMR status of surgical pathologic specimens. According to the guidelines of NCCN and the Chinese Society of Clinical Oncology, if the expression of MMR gene-related proteins (MLH1, MSH2, MSH6, and PMS2) are lacking in immunohistochemical detection, it will be defined as dMMR. The MMR protein deficiency of patients is shown in Fig. 1C. When the IHC result was uncertain, it was further confirmed by the microsatellite instability (MSI) test based on PCR. The pathologic staging of the CRC patients was determined according to the International Union Against Cancer tumor-node-metastasis (TNM) staging/American Joint Committee on Cancer staging.

### Statistical Analysis

GraphPad Prism 9 (San Diego, CA, USA) and SPSS 25.0 (New York, NY, USA) were used for data analysis in this study. The analysis results showed that the distribution of mtDNA-CN was skewed toward the right (Shapiro-Wilk’s W, 0.9231; *P* < 0.0001), which did not conform to the normal distribution (Fig. 1B). Therefore, we adopted non-parametric statistical test methods for data analysis. The Mann-Whitney’s *U* test and the Kruskal-Wallis test were used to analyze the relationship between mtDNA-CN and clinicopathologic features where applicable. The chi-square test was used to analyze the classification parameters, and the number and percentage of cases were recorded.

For comparison of continuous variables and means, the Mann-Whitney *U* test or Student’s *t* test was used. Regarding survival-related data, the Kaplan-Meier log-rank test was used to compare the effects of various factors on the DFS and OS of the patients. Moreover, the potential prognostic factors were analyzed by the univariate Cox model, and the statistically significant parameters were further determined by the multivariate Cox model. In view of the interaction between indicators and ACT, the Cox proportional hazards regression model was used. The output results included hazard ratios (HRs) and 95% confidence interval (CIs). For all data analysis results, when the two-sided *P* value was lower than 0.05, we considered the difference as statistically significant.

## Results

### Characteristics of the CRC Patients With the dMMR Cohort

This study encompassed 308 eligible CRC patients, comprising 180 with stage II and 128 with stage III disease. The age of the participants ranged from 17 to 89 years (median, 54 years). Among the analyzed cohort, 195 (63.3%) were male, and 113 (36.7%) were female. After surgery, 197 patients (64.0%) received ACT, whereas 111 patients (36.0%) did not. During a median follow-up period of 80 months, 63 patients (20.5%) experienced disease recurrence, and 42 patients (13.6%) succumbed to CRC. The clinicopathologic characteristics and treatment details for the cohort of CRC patients with dMMR are presented in Table 1.

**Table 1:** Clinicopathologic features and treatment of stages II and III colorectal cancer patients with deficient mismatch repair

Characteristics	*n* (%)	Characteristics	*n* (%)
Age (years)		Tissue type	
<65	240 (77.9)	Adenocarcinoma	240 (77.9)
≥65	68 (22.1)	Mucinous adenocarcinoma	68 (22.1)
Gender		Tumor differentiation	
Male	195 (63.3)	Poor/moderate	222 (72.1)
Female	113 (36.7)	Well	86 (27.9)
Distance from anal verge (cm)		CEA (ng/mL)	
≤5	49 (15.9)	<5	221 (71.8)
>5	259 (84.1)	≥5	87 (28.2)
Preoperative therapy		CA19-9 (U/mL)	
No	247 (80.2)	<37	246 (79.9)
Yes	61 (19.8)	≥37	62 (20.1)
Pathologic T stage		Lymphovascular invasion	
3	255 (82.8)	–	263 (85.4)
4	53 (17.2)	+	45 (14.6)
Pathologic N stage		Nervous invasion	
0	180 (58.4)	–	286 (92.9)
1–2	128 (41.6)	+	22 (7.1)
Tumor location		Adjuvant chemotherapy	
Colon	234 (76.0)	No	111 (36.0)
Rectum	74 (24.0)	Yes	197 (64.0)

*CEA*, Carcinoembryonic antigen; CA19-9, Glucose carbohydrate antigen 19-9

### Correlation of mtDNA-CN With Clinicopathologic Features and Prognosis

**Fig. 2:**
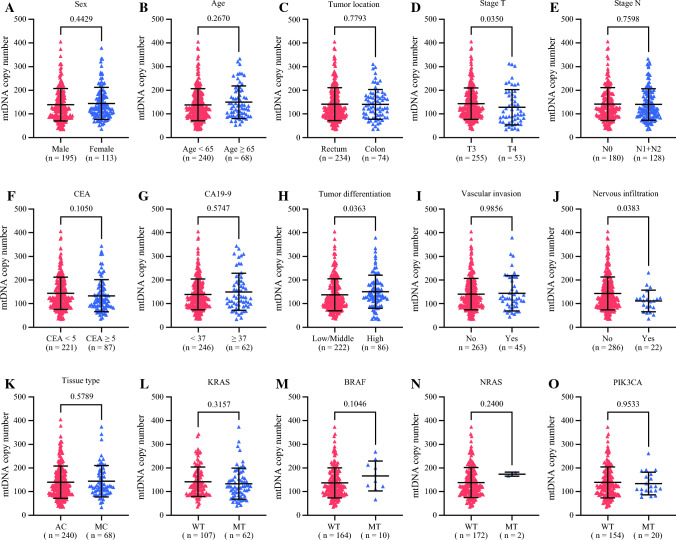
Correlations between mtDNA-CN and clinicopathologic features in stages II and III CRC with dMMR. **A** Sex. **B** Age. **C** Tumor location. **D** Stage T. **E** Stage N. **F** CEA. **G** CA19-9. **H** Tumor differentiation. **I** Vascular invasion. **J** Nervous infiltration. **K** Tissue type. **L** KRAS status. **M** BRAF status. **N** NRAS status. **O** PIK3CA status. MtDNA-CN, mitochondrial DNA copy number; CRC, colorectal cancer; dMMR, deficient mismatch repair; CEA, carcinoembryonic antigen; CA 19-9, carbohydrate antigen 19-9; AC, adenocarcinoma; MC, mucinous adenocarcinoma; WT, wild type; MT, mutant type

**Fig. 3:**
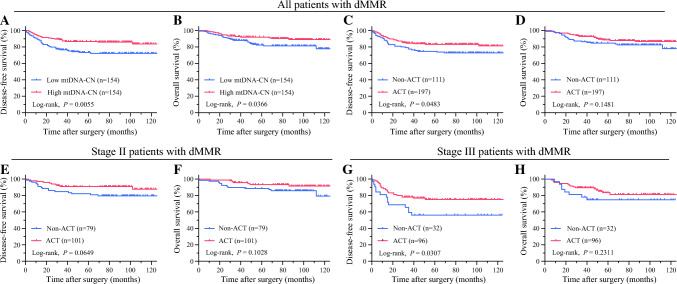
Correlation between ACT, mtDNA-CN and the prognosis of stages II and III CRC patients with dMMR. **A** Kaplan-Meier analysis of DFS based on mtDNA-CN in the CRC cohort. **B** Kaplan-Meier analysis of OS based on mtDNA-CN in the CRC cohort. **C** Kaplan-Meier analysis of DFS based on ACT in the CRC cohort. **D** Kaplan-Meier analysis of OS based on ACT in the CRC cohort. **E** Kaplan-Meier analysis of DFS based on ACT in the stage II CRC with dMMR cohort. **F** Kaplan-Meier analysis of OS based on ACT in the stage II CRC with dMMR cohort. **G** Kaplan-Meier analysis of DFS based on ACT in the stage III CRC with dMMR cohort. **H** Kaplan-Meier analysis of OS based on ACT in the stage III CRC with dMMR cohort. ACT, adjuvant chemotherapy; MtDNA-CN, mitochondrial DNA copy number; CRC, colorectal cancer; dMMR, deficient mismatch repair; DFS, disease-free survival; OS, overall survival

Subsequently, we performed an analysis of the distribution and prognostic implications of mtDNA-CN. Notably, mtDNA-CN exhibited a statistically significant reduction in tumor tissues compared with non-cancerous tissues (*P* < 0.0001; Fig. 1D). Specifically, an elevation in mtDNA-CN was prominently observed in cases classified as stage T4 (*P* = 0.0350; Fig. 2D),<F2> exhibiting poor tumor differentiation (*P* = 0.0363; Fig. 2H) and manifesting nervous infiltration (*P* = 0.0383; Fig. 2J). Conversely, no statistically significant associations were identified between mtDNA-CN and variables such as sex (*P* = 0.4429), age (*P* = 0.2670), tumor location (*P* = 0.7793), stage N (*P* = 0.7598), CEA (*P* = 0.1050), carbohydrate antigen 19-9 (CA19-9) (*P* = 0.5747), vascular invasion (*P* = 0.9856), tissue type (*P* = 0.5789), KRAS status (*P* = 0.3157), BRAF status (*P* = 0.1046), NRAS status (*P* = 0.2400), and PIK3CA status (*P* = 0.9533). Furthermore, we observed a correlation between low mtDNA-CN and unfavorable disease-free survival (DFS) (73.4% vs 85.7%, *P* = 0.0055) and overall survival (OS) (82.5% vs 90.3%, *P* = 0.0366) in the CRC patients with dMMR (Fig. 3A and B).

### Prognostic Analysis

To elucidate the prognostic significance of mtDNA-CN, Cox regression analysis was performed on all the CRC patients with dMMR. Univariate analysis showed that age older than 65 years, pathologic T stage 4, positive pathologic N stage, lymphovascular infiltration, and nervous invasion were associated with poorer DFS for the CRC patients with dMMR. Additionally, elevated mtDNA-CN emerged as a favorable factor for DFS. In the multivariate analysis, age older than 65 years (HR 2.073, 95% CI 1.233–3.488; *P* = 0.0059), lymphovascular infiltration (HR 2.360; 95% CI 1.320–4.217; *P* = 0.0037), nervous invasion (HR 2.557; 95% CI 1.327–4.926; *P* = 0.0050), and high mtDNA-CN (HR 0.547; 95% CI 0.321–0.934; *P* = 0.0270) were identified as independent factors influencing DFS (Table S1).

Furthermore, the univariate analysis demonstrated that age older than 65 years, pathologic T stage 4, positive pathologic N stage, lymphovascular infiltration, and nervous invasion were correlated with poorer OS. In addition, elevated mtDNA-CN emerged as a favorable factor for OS. The multivariate analysis confirmed that age older than 65 years (HR 3.634; 95% CI 1.958–6.745; *P* < 0.0001) and high mtDNA-CN (HR 0.520; 95% CI 0.272–0.998; *P* = 0.0492) were independent factors influencing OS (Table S2).

### The Role of Adjuvant Chemotherapy for the CRC Patients With dMMR

Regarding the impact of ACT on the prognosis for the CRC patients with dMMR, the utilization of ACT exhibited a significant enhancement in DFS (73.0% vs 83.2%; *P* = 0.0483), whereas it did not demonstrate a statistically significant effect on OS (82.0% vs 88.8%; *P* = 0.1481) (Fig. 3C and D). In the subgroup analysis focused on stage II CRC patients with dMMR, ACT did not yield a significant impact on DFS (79.7% vs 90.1%; *P* = 0.0649) or OS (84.8% vs 93.1%; *P* = 0.1028) (Fig. 3E and F). Conversely, for the stage III CRC patients with dMMR, ACT significantly improved DFS (56.3% vs 76.0%; *P* = 0.0307), although it did not exert a significant effect on OS (75.0% vs 84.4%; *P* = 0.2311) (Fig. 3G and H). Cox regression analysis indicated that in the broader context of all the CRC patients with dMMR, ACT did not emerge as a significant prognostic factor for DFS (HR 0.611; 95% CI 0.373–1.002; *P* = 0.0510) or OS (HR 0.642; 95% CI 0.350–1.177; *P* = 0.1518) (Tables S1 and S2).

### MtDNA-CN as a Prognostic Biomarker for the Efficacy of Adjuvant Chemotherapy

**Fig. 4:**
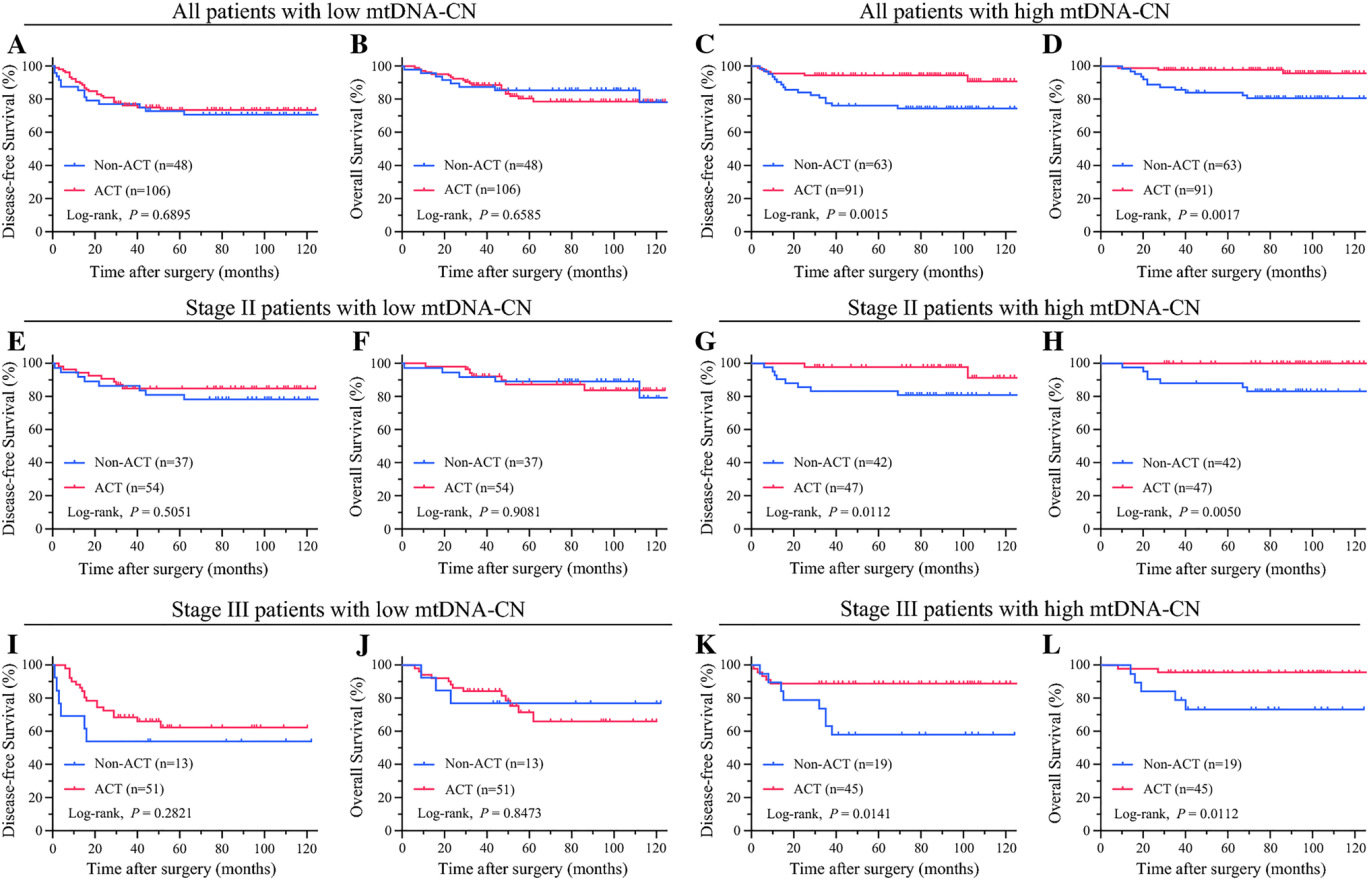
A subgroup analysis of the correlation between ACT, mtDNA-CN, and the prognosis of stages II and III CRC patients with dMMR. **A** Kaplan-Meier analysis of DFS based on ACT in the low mtDNA-CN CRC cohort. **B** Kaplan-Meier analysis of OS based on ACT in the low mtDNA-CN CRC cohort. **C** Kaplan-Meier analysis of DFS based on ACT in the high mtDNA-CN CRC cohort. **D** Kaplan-Meier analysis of OS based on ACT in the high mtDNA-CN CRC cohort. **E** Kaplan-Meier analysis of DFS based on ACT in the stage II dMMR CRC with low mtDNA-CN cohort. **F** Kaplan-Meier analysis of OS based on ACT in the stage II dMMR CRC with low mtDNA-CN cohort. **G** Kaplan-Meier analysis of DFS based on ACT in the stage II dMMR CRC with high mtDNA-CN cohort. **H** Kaplan-Meier analysis of OS based on ACT in the stage II dMMR CRC with high mtDNA-CN cohort. **I** Kaplan-Meier analysis of DFS based on ACT in the stage III dMMR CRC with low mtDNA-CN cohort. **J** Kaplan-Meier analysis of OS based on ACT in the stage III dMMR CRC with low mtDNA-CN cohort. **K** Kaplan-Meier analysis of DFS based on ACT in the stage III dMMR CRC with high mtDNA-CN cohort. **L** Kaplan-Meier analysis of OS based on ACT in the stage III dMMR CRC with high mtDNA-CN cohort. ACT, adjuvant chemotherapy; MtDNA-CN, mitochondrial DNA copy number; CRC, colorectal cancer; dMMR, deficient mismatch repair; DFS, disease-free survival; OS, overall survival

We further investigated the potential of mtDNA-CN as a predictive biomarker for the efficacy of ACT for patients with dMMR. Among the dMMR CRC patients with low mtDNA-CN, ACT demonstrated no significant impact on DFS (70.8% vs 74.5%; *P* = 0.6895) or OS (83.3% vs 82.1%; *P* = 0.6585) (Fig. 4A and B). Conversely, for the dMMR CRC patients with high mtDNA-CN, ACT significantly improved both DFS (74.6% vs 93.4%; *P* = 0.0015) and OS (81.0% vs 96.7%; *P* = 0.0017) (Fig. 4C and D).

To assess whether staging influences outcomes, we conducted subgroup analyses of both staging and mtDNA-CN. For the stage II dMMR CRC patients with low mtDNA-CN, ACT had no discernible effect on DFS (78.4% vs 85.2%; *P* = 0.5051) or OS (86.5% vs 87.0%; *P* = 0.9081) (Fig. 4E and F). In contrast, for the stage II dMMR CRC patients with high mtDNA-CN, ACT significantly improved both DFS (81.0% vs 95.7%; *P* = 0.0112) and OS (83.3% vs 100.0%; *P* = 0.0050) (Fig. 4G and H).

Similarly, for the stage III dMMR CRC patients with high mtDNA-CN, ACT significantly enhanced both DFS (57.9% vs 88.9%; *P* = 0.0141) and OS (73.7% vs 95.6%; *P* = 0.0112) (Fig. 4K and L). Conversely, for the stage III dMMR CRC patients with low mtDNA-CN, ACT had no apparent effect on DFS (53.8% vs 64.7%; *P* = 0.2821) or OS (76.9% vs 74.5%; *P* = 0.8473) (Fig. 4I and J).

In addition, subgroup analyses for tumor location showed that tumor location did not appear to influence these findings (Fig. S1). Given the limited subgroup sample size, the results need to be analyzed with caution.

Notably, inherent differences in baseline characteristics exist between patients with and without ACT. To delve deeper into the relationship between mtDNA-CN and ACT, we performed subgroup analyses on all the dMMR CRC patients who received ACT. These analyses showed that for all the dMMR CRC patients with ACT, those who had high mtDNA-CN exhibited superior DFS (73.5% vs 92.9%; *P* = 0.0003) and OS (80.6% vs 97.0%; *P* = 0.0001) (Fig. S2A and B).<SF2> Additionally, among the stage II dMMR CRC patients with ACT, those with high mtDNA-CN showed improved DFS (84.0% vs 96.1%; *P* = 0.0400) and OS (88.0% vs 98.0%; *P* = 0.0423) (Fig. S2C and D). Similarly, among the stage III dMMR CRC patients with ACT, those with high mtDNA-CN also demonstrated enhanced DFS (62.5% vs 89.6%; *P* = 0.0030) and OS (72.9% vs 95.8%; *P* = 0.0008) (Fig. S2E and F).

### Predictive Effect of Various Factors on the Efficacy of Adjuvant Chemotherapy

**Fig. 5:**
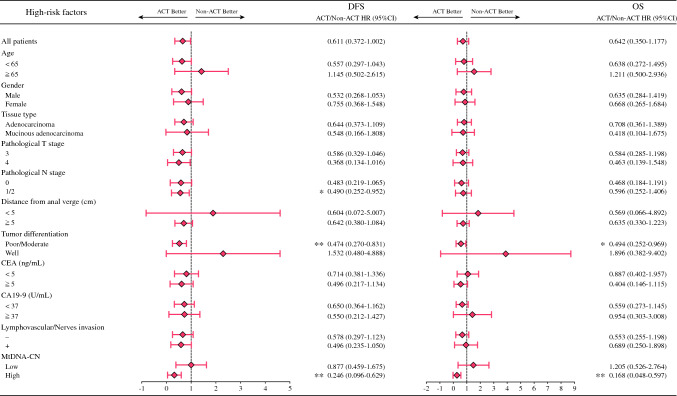
Efficacy of ACT on each clinicopathologic factor and mtDNA-CN for DFS and OS for stages II and III CRC patients with dMMR. ACT, adjuvant chemotherapy; MtDNA-CN, mitochondrial DNA copy number; DFS, disease-free survival; OS, overall survival; CRC, colorectal cancer; dMMR, deficient mismatch repair; CEA, carcinoembryonic antigen; CA 19-9, carbohydrate antigen 19-9

To assess the superiority of mtDNA-CN over other clinicopathologic factors in predicting the efficacy of ACT for CRC patients with dMMR, all relevant factors were evaluated. Regarding DFS, ACT conferred survival benefit for the dMMR CRC patients with positive pathologic N stage (HR 0.490; 95% CI 0.252–0.952; *P* = 0.0353), poor tumor differentiation (HR 0.474; 95% CI 0.270–0.831; *P* = 0.0092), and high mtDNA-CN (HR 0.246; 95% CI 0.096–0.629; *P* = 0.0034) (Fig. 5). Regarding OS, ACT conferred a survival benefit for the dMMR CRC patients with poor tumor differentiation (HR 0.494; 95% CI 0.252–0.969; *P* = 0.0402) and high mtDNA-CN (HR 0.168; 95% CI 0.048–0.597; *P* = 0.0058) (Fig. 5). These results suggest that among all clinicopathologic factors, high mtDNA-CN stands out as the most effective predictor of ACT efficacy.

## Discussion

This study represents the inaugural disclosure of mtDNA-CN as a potential predictive biomarker for the efficacy of ACT for CRC patients with dMMR. Specifically, for the stage II/III dMMR CRC patients with elevated mtDNA-CN, the administration of ACT significantly improved both DFS and OS. This discovery lays a crucial foundation for tailoring individualized ACT treatment strategies for CRC patients with dMMR, highlighting the significant role of mtDNA-CN in guiding treatment decisions.

In recent years, there has been a growing consensus among medical professionals that MMR status serves as a practical biomarker to inform postoperative treatment decisions in advanced CRC.^21^ The current NCCN guidelines recommend against ACT for stage II CRC patients with dMMR/MSI-H while advocating for ACT for stage III CRC patients with dMMR/MSI-H.^14^ This approach is considered relatively safe due to ongoing debates surrounding the use of ACT for CRC patients with dMMR. Numerous clinical data indicate a potential diminished response to fluorouracil in dMMR CRC patients.^8,9^ Furthermore, fluorouracil-based ACT may even compromise the survival of CRC patients with dMMR.^22^

These clinical findings align with in vitro experimental results, which demonstrate increased resistance of MSI CRC cells to fluorouracil.^23^ In fact, a competent MMR system is crucial for the efficacy of fluorouracil because MMR proteins promote DNA damage-induced apoptosis.^24^ Therefore, it has been suggested that CRC patients with dMMR should not undergo adjuvant fluorouracil therapy. However, this conclusion is not universally accepted, and several studies have reported inconclusive findings regarding the benefits or harms of ACT for CRC patients with dMMR.^25,26^

In contrast, the addition of oxaliplatin to fluorouracil may confer a survival advantage for stage III CRC patients with dMMR.^11,21^ A study by Shaib et al.^27^ demonstrated a significant OS benefit for stage III CRC patients with dMMR who received ACT, particularly with doublet chemotherapy. Retrospective analyses from various clinical trials have indicated that folinic acid-fluorouracil-oxaliplatin (FOLFOX) treatment surpasses fluorouracil alone in efficacy for dMMR CRC.^28,29^ Preclinical data have similarly indicated that MSI tumor cell lines exhibit sensitivity to oxaliplatin.^30^ Unlike fluorouracil, oxaliplatin achieves its chemotherapeutic effect through the formation of platinum-DNA adducts, causing DNA damage, including mtDNA, leading to apoptosis, a process not recognized by the MMR system.^31,32^ However, Müller et al.’s^12^ findings suggested that MSI-H metastatic CRC patients were less responsive than MSS patients to 5-fluorouracil/folinic acid plus oxaliplatin (FUFOX) or capecitabine plus oxaliplatin (CAPOX) regimens, implying a potential resistance of dMMR status to oxaliplatin-based chemotherapy. Conversely, Kim et al.^13^ argued that MMR status has no significant impact on the prognosis for patients who receive oxaliplatin-based chemotherapy.^13^

In summary, the therapeutic efficacy of ACT for CRC patients with dMMR exhibits variability, indicating treatment response heterogeneity in this subgroup. Consequently, sensitivity to ACT may vary among CRC patients with dMMR, warranting the need for individualized treatment due to cost implications, concomitant toxicity, and limited survival benefit.

Identifying a novel biomarker for predicting the response of CRC patients with dMMR to ACT is crucial. Notably, prior studies by Duval’s team suggested that the large deletion of HSP110 T_17_ correlated with ACT efficacy, either with fluorouracil alone or combined with oxaliplatin, in CRC patients with dMMR.^33^ Regrettably, this effect was not consistently observed in subsequent studies.^34,35^

Another investigation by Ryan et al.^36^ explored the impact of COX2 on the ACT response in CRC patients with dMMR but found no discernible relationship. Our study, in contrast, showed the adverse prognosis associated with low mtDNA-CN in CRC patients with dMMR. Importantly, our results provide evidence that mtDNA-CN levels may help identify a subset of stage II or III CRC patients with dMMR who could benefit from ACT, suggesting mtDNA-CN as a potential predictive biomarker for ACT efficacy.

As a potential biomarker, mtDNA-CN has demonstrated associations with various diseases and cancers.^37^ Additionally, mitochondrial dysfunction, resulting from genetic changes in mtDNA, has been closely linked to chemoresistance.^17,19^ In ovarian cancer, depleted mtDNA-CN suppresses production of reactive oxygen species, leading to cisplatin insensitivity.^19^ Furthermore, in esophageal squamous cancer cells, low mtDNA-CN induces resistance to cisplatin, fluorouracil, and docetaxel chemotherapy via epithelial-mesenchymal transition.^18^ Previous studies also have indicated that MSI-H CRC with a high frequency of TFAM-truncating mutations demonstrates insensitivity to cisplatin-induced apoptosis due to increased cytochrome b expression.^20^ Therefore, these findings collectively suggest that mtDNA-CN holds promise as a predictive biomarker for ACT efficacy. From a clinical perspective, the mtDNA-CN test based on qRT-PCR can be performed easily in most hospital laboratories and is cost-effective.

Admittedly, this study had some limitations. First, due to the retrospective design of the study, the lack of some clinical information may have affected the richness and completeness of the results. Second, the patient cohort was not continuously collected, and the sample size was relatively limited, which may have affected the representativeness of the research results. Third, due to the limited sample size, the independent analysis results of colon cancer and rectal cancer could not present potential differences well. Fourth, the predictive efficacy of mtDNA-CN lacks external verification, which limits the generalization of the research results. Finally, this study was conducted within a particular demographic and genetic background, which might limit the applicability of the findings to broader populations. Therefore, we hope that more rigorous prospective studies will be conducted in the future to verify the predictive effect of mtDNA-CN on the acceptance of ACT for CRC patients with dMMR, so the purpose of “seeking benefits and avoiding harms” can be achieved.

## Conclusion

In conclusion, our study presents compelling evidence supporting the potential utility of mtDNA-CN levels for identifying a subset of stage II/III CRC patients with dMMR who could derive benefits from ACT. This insight underscores the significance of personalized treatment strategies. The assessment of mtDNA-CN through qRT-PCR emerges as a straightforward and feasible method, offering practical applicability in clinical settings.

### Supplementary Information

Below is the link to the electronic supplementary material.

Supplementary file1 (DOCX 483 kb)

## Data Availability

All the data are available from the corresponding author on reasonable request.
