# Supplementary material for: Mitochondrial DNA Copy Number as a Biomarker for Guiding Adjuvant Chemotherapy in Stages II and III Colorectal Cancer Patients with Mismatch Repair Deficiency: Seeking Benefits and Avoiding Harms
Source: Ann Surg Oncol. 2024 Jul 10;31(9):6320–30. doi: 10.1245/s10434-024-15759-y (PMC11300489; doi:10.1245/s10434-024-15759-y)

**Fig. S1** A subgroup analysis of the correlation between tumor location, ACT, mtDNA-CN, and prognosis for stages II and III colon patients with dMMR. **A** Kaplan-Meier analysis of DFS based on ACT in the low mtDNA-CN colon cancer cohort. **B** Kaplan-Meier analysis of OS based on ACT in the low mtDNA-CN colon cancer cohort. **C** Kaplan-Meier analysis of DFS based on ACT in the high mtDNA-CN colon cancer cohort. **D** Kaplan-Meier analysis of OS based on ACT in the high mtDNA-CN colon cancer cohort. **E** Kaplan-Meier analysis of DFS based on ACT in the low mtDNA-CN rectal cancer cohort. **F** Kaplan-Meier analysis of OS based on ACT in the low mtDNA-CN rectal cancer cohort. **G** Kaplan-Meier analysis of DFS based on ACT in the high mtDNA-CN rectal cancer cohort. **H** Kaplan-Meier analysis of OS based on ACT in the high mtDNA-CN rectal cancer cohort. MtDNA-CN, mitochondrial DNA copy number; dMMR, deficient mismatch repair; ACT, adjuvant chemotherapy; DFS, disease-free survival; OS, overall survival

**Fig. S2** Correlation between mtDNA-CN and the prognosis of stages II and III dMMR CRC patients with ACT. **A** Kaplan-Meier analysis of DFS based on mtDNA-CN in the CRC cohort. **B** Kaplan-Meier analysis of OS based on mtDNA-CN in the CRC cohort. **C** Kaplan-Meier analysis of DFS based on ACT in the stage II CRC cohort. **D** Kaplan-Meier analysis of OS based on ACT in the stage II CRC cohort. **E** Kaplan-Meier analysis of DFS based on ACT in the stage III CRC with dMMR cohort. **F** Kaplan-Meier analysis of OS based on ACT in the stage III CRC with dMMR cohort. MtDNA-CN, mitochondrial DNA copy number; dMMR, deficient mismatch repair; CRC, colorectal cancer; ACT, adjuvant chemotherapy; DFS, disease-free survival; OS, overall survival

**TABLE S1** Cox regression analysis of prognostic factors for the DFS of stages II and III colorectal cancer patients with deficient mismatch repair

| Factors | Univariate analysis | | Multivariate analysis | |
| --- | --- | --- | --- | --- |
|  | HR (95 % CI) | *P* Value | HR (95 % CI) | *P* Value |
| Age >65 years | 2.132 (1.276–3.561) | 0.0038 | 2.073 (1.233–3.488) | 0.0059 |
| Gender (female) | 1.616 (0.986–2.650) | 0.0570 |  |  |
| Distance from anal verge >5 cm | 1.505 (0.686–3.305) | 0.3079 |  |  |
| Tissue type (MC) | 0.722 (0.377–1.384) | 0.3270 |  |  |
| Pathologic T stage 4 | 2.075 (1.188–3.623) | 0.0102 | 1.709 (0.965–3.025) | 0.0659 |
| Pathological N stage positive | 2.288 (1.384–3.783) | 0.0012 | 1.631 (0.945–2.816) | 0.0789 |
| CEA >5 ng/mL | 1.590 (0.952–2.657) | 0.0764 |  |  |
| CA19-9 >37 U/mL | 1.612 (0.924–2.812) | 0.0927 |  |  |
| Poor tumor differentiation | 1.483 (0.819–2.687) | 0.1937 |  |  |
| Lymphovascular infiltration | 3.264 (1.904–5.596) | < 0.0001 | 2.360 (1.320–4.217) | 0.0037 |
| Nervous invasion | 3.933 (2.092–7.395) | < 0.0001 | 2.557 (1.327–4.926) | 0.0050 |
| Adjuvant chemotherapy | 0.611 (0.373–1.002) | 0.0510 |  |  |
| High mtDNA-CN | 0.488 (0.291–0.819) | 0.0066 | 0.547 (0.321–0.934) | 0.0270 |

DSF, disease-free survival; HR, hazard ratio; CI, confidence interval; MC, mucinous adenocarcinoma; CEA, carcinoembryonic antigen; CA19-9, glucose carbohydrate antigen 19-9; MtDNA-CN, mitochondrial DNA copy number.

**TABLE S2** Cox regression analysis of prognostic factors for the OS of stages II and III colorectal cancer patients with deficient mismatch repair

| Factors | Univariate analysis | | Multivariate analysis | |
| --- | --- | --- | --- | --- |
|  | HR (95 % CI) | *P* Value | HR (95 % CI) | *P* Value |
| Age >65 years | 3.513 (1.917–6.439) | <0.0001 | 3.634 (1.958–6.745) | <0.0001 |
| Gender (female) | 1.270 (0.689–2.341) | 0.4437 |  |  |
| Distance from anal verge >5 cm | 1.065 (0.448–2.529) | 0.8871 |  |  |
| Tissue type (MC) | 0.873 (0.404–1.888) | 0.7305 |  |  |
| Pathologic T stage 4 | 2.309 (1.179–4.522) | 0.0146 | 1.834 (0.898–3.746) | 0.0959 |
| Pathologic N stage positive | 1.958 (1.064–3.603) | 0.0308 | 1.616 (0.830–3.144) | 0.1580 |
| CEA >5 ng/mL | 1.648 (0.883–3.074) | 0.1163 |  |  |
| CA19-9 >37 U/mL | 1.679 (0.860–3.280) | 0.1291 |  |  |
| Poor tumor differentiation | 1.825 (0.844–3.946) | 0.1262 |  |  |
| Lymphovascular infiltration | 2.462 (1.236–4.906) | 0.0104 | 1.704 (0.812–3.576) | 0.1590 |
| Nervous invasion | 2.713 (1.200–6.135) | 0.0164 | 1.426 (0.587–3.463) | 0.4331 |
| Adjuvant chemotherapy | 0.642 (0.350–1.177) | 0.1518 |  |  |
| High mtDNA-CN | 0.516 (0.274–0.971) | 0.0403 | 0.520 (0.272–0.998) | 0.0492 |

OS, overall survival; HR, hazard ratio; CI, confidence interval; MC, mucinous adenocarcinoma; CEA, carcinoembryonic antigen; CA19-9, glucose carbohydrate antigen 19-9; MtDNA-CN, mitochondrial DNA copy number.


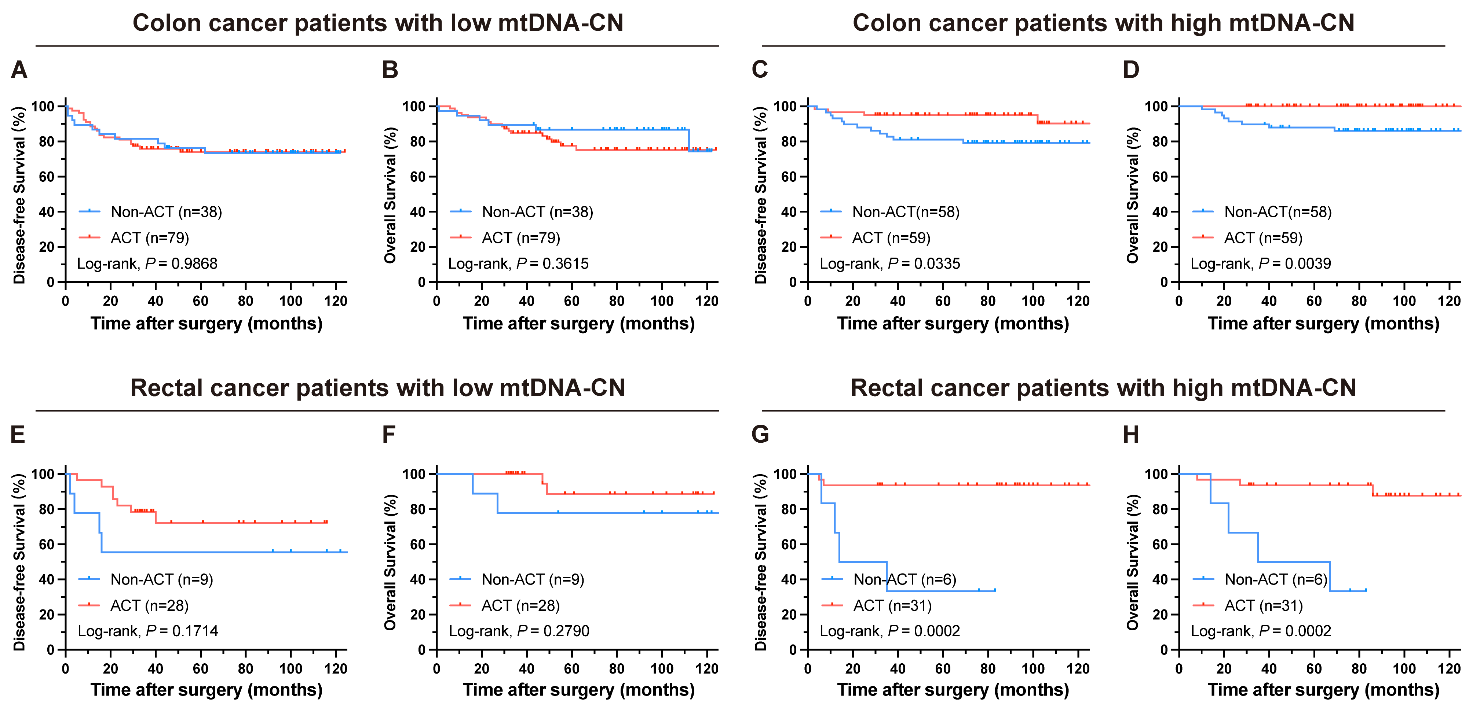

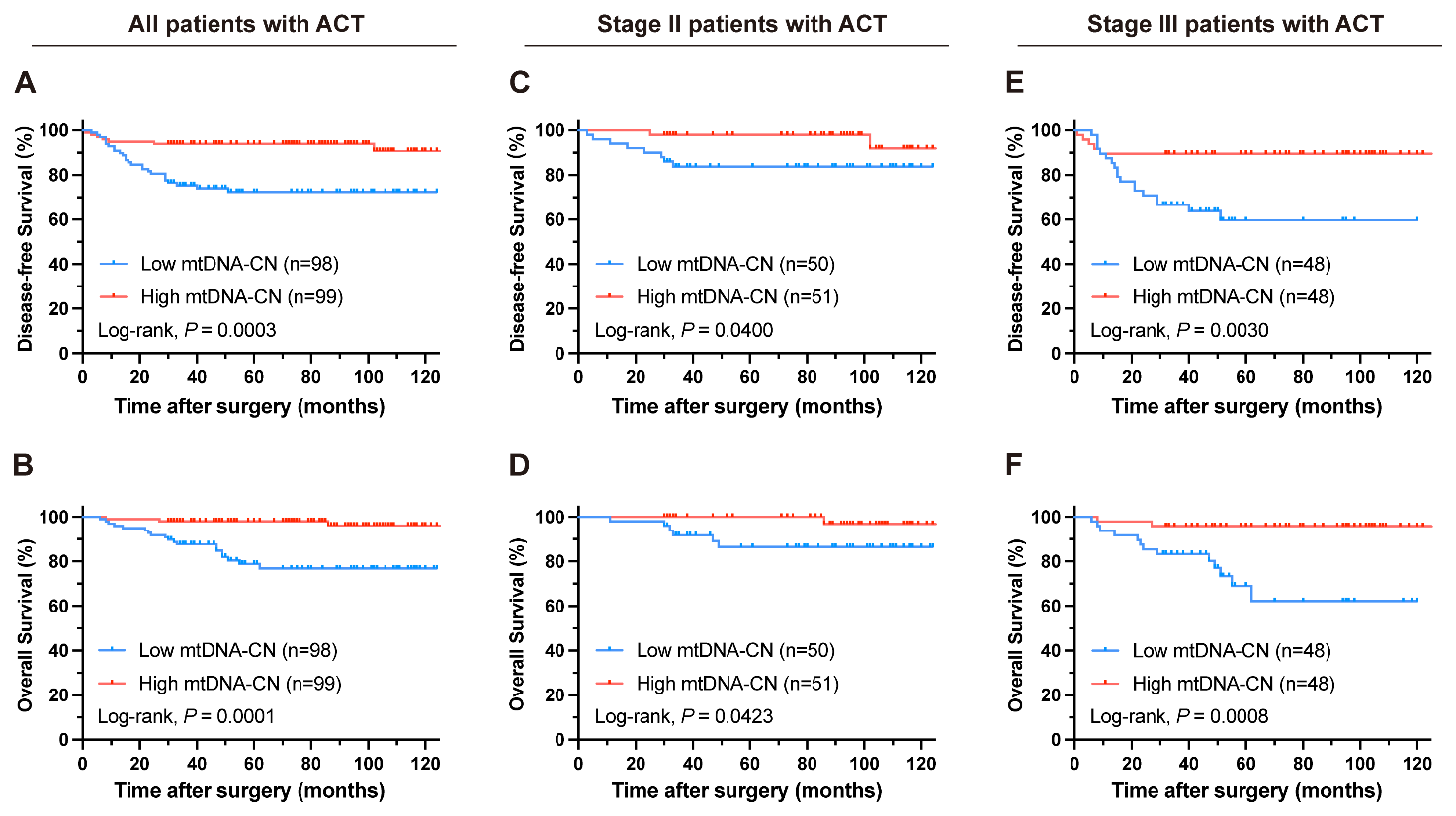

Supplement: Supplementary file 1 — Supplementary file1 (DOCX 483 kb) [file 10434_2024_15759_MOESM1_ESM.docx]
